# Synthesis of Lignocellulose-Based Poly(Butylene 3-Propyladipate-Co-Furanoate): Replacing Adipate

**DOI:** 10.3390/molecules30040878

**Published:** 2025-02-14

**Authors:** Ruijia Hu, Weihao Li, Xi Zhou, Shunmin Yi, Xingfu Zheng, Qiufeng Mo, Wanyu Liu

**Affiliations:** 1 Guangxi Key Laboratory of Advanced Microwave Manufacturing Technology, Guangxi Academy of Sciences, Nanning 530007, China; lwh17877259373@163.com (W.L.); zx_gxkx@163.com (X.Z.); zhengxingfu@gxas.cn (X.Z.); 18078131362@163.com (Q.M.); 2 Guangxi Key Laboratory of Advanced Structural Materials and Carbon Neutralization, Guangxi Colleges and Universities Key Laboratory of Environmental-Friendly Materials and Ecological Restoration, School of Materials and Enviroment, Guangxi Minzu University, Nanning 530105, China; 20200040@gxmzu.edu.cn

**Keywords:** lignocellulose, melt polymerization, copolyesters, poly(butylene 3-propyladipate-co-furanoate)

## Abstract

A novel lignocellulose-based poly(butylene 3-propyladipate-co-furanoate) (PBA_p_F) was synthesized from lignocellulose-derived 3-propyladipic acid (3PAA), 1,4-butanediol (BDO), and 2,5-furandicarboxylic acid (FDCA). The copolyesters were characterized by ^1^H NMR, GPC, DSC, TGA, XRD, DMA, rotational rheology, tensile tests, and enzymatic degradation tests. They were random copolymers whose composition was well controlled, and the number-average sequence lengths of the copolyesters were around 1.35–4.33. By combining the results of tensile tests and DMA, the elongation at break of PBA_p_F_45_ (1865%) had a much greater value than that of PBAF_45_ (1250%), i.e., the branching incorporated into the linear polymer increased the melt strength and conferred tension-hardening properties, which helped to enhance the elongation of the polymer. In addition, the influences of 3PAA content on enzymatic degradation were studied in terms of weight loss; when the content of 3PAA was 55 mol%, the copolyesters exhibited good biodegradability. Thus, depending on their composition, PBA_p_F_S_ might find end applications as biodegradable elastomers or impact modifiers for other polymers.

## 1. Introduction

The current polymer industries are immensely dependent on petroleum resources. With the reduction in the oil stocks in the world and the emergence of environmental problems, it is very important to develop alternatives to petroleum-based polymer materials. Bio-based polymer materials have been considered to be the most promising alternative and are undergoing a number of studies [1,2,3]. Polymers using renewable resources as terminal feedstocks are crucial to the sustainable chemical industry [4,5,6,7,8]. Carbohydrates are the most abundant source of renewable materials on earth. Among these, furan derivatives are important raw materials for the preparation of polyesters or polyamides [1,9,10,11]. The United States (US) Department of Energy has evaluated FDCA to be one of the priority chemicals that play an important role in the green chemical industry [12,13].

Petroleum-based terephthalic acid (TPA) is used to produce important commodity polyesters such as poly(ethylene terephthalate) (PET), poly(butylene terephthalate) (PBT), and poly(butylene adipate-co-butylene terephthalate) (PBAT). FDCA is a bio-based aromatic diacid monomer, which can be obtained from biomass [14,15,16,17] and used to prepare furan-based polyesters [18,19,20,21,22]. For example, it can be produced from cellulose or hemicelluloses through a multi-step process, including bioconversion, dehydration, and oxidation in which hexose and 5-hydroxymethyl furfural are important intermediates [3,23,24]. Because its structural, chemical, and physical properties are similar to TPA, considerable work has focused on the development of FDCA-based polyester in order to replace TPA-based polyester [25,26,27,28,29,30].

Semi-aromatic polyesters combine the high melting points and heat resistance of wholly aromatic polyesters and the good melt processability of aliphatic polyesters. PBAT has successfully achieved an adjustable balance between biodegradation and physical properties [31,32], and can be used in food packaging materials [33], mulching film [34], and biomedical materials [35]. It is a semi-aromatic copolyester whose crystallinity, mechanical properties, and biodegradability can be controlled by the composition of aliphatic and aromatic contents [36,37]. For example, PBAT with good tear resistance, flexibility, and water resistance can be prepared by keeping the content of aromatics and aliphatics at a ratio of 50:50 [38]. However, PBAT is a petroleum-based polyester.

Weidong Zhou and co-workers [39] synthesized a series of poly (butylene adipate-co-furanoate) (PBAF) materials using a two-step polycondensation reaction, and studied the tensile tests and enzymatic degradation of copolyesters. According to the composition, the PBAF copolyesters showed characteristics from thermoplastics to elastomers. At a lower FDCA content, the amorphous copolyesters had a low glass transition temperature (*T*_g_) and high elongation; at a higher FDCA content, the semi-crystalline copolyesters had high Tm and strength. In addition, the biodegradability of the copolyesters could be controlled by adjusting the content of FDCA. Hyeri and co-workers [40] synthesized an FDCA-based polyester (PBAF), which showed excellent mechanical properties and degradability (FDCA ester groups trigger enzymatic hydrolysis). Thermal studies of PBAF revealed the influences of structure and composition on crystallization and melting behaviors. Rheological analyses confirmed that asymmetric and semi-rigid FDCA-based PBAF experienced greater chain entanglement than rigid TPA-based PBAT, ultimately developing stronger elasticity [40].

In this study, a novel lignocellulose-based PBA_p_F was synthesized from lignocellulose-derived 3PAA, BDO, and FDCA. The copolyesters were characterized by ^1^H NMR, GPC, DSC, TGA, XRD, DMA, rotational rheology, tensile tests, and enzymatic degradation tests to reveal the effects of branched 3PAA on the properties of copolyesters.

## 2. Results and Discussion

### 2.1. Chain Structure and Sequence of Copolyesters

**Figure 1:**
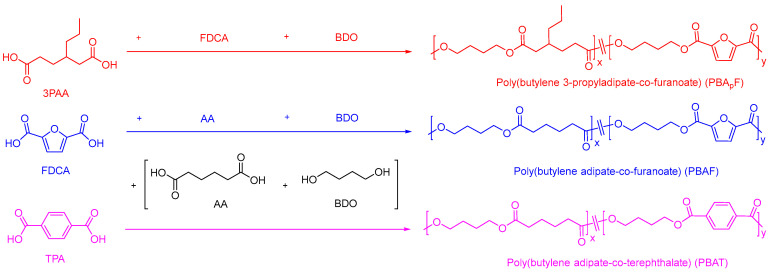
Polymerization procedure for two series of copolyesters.

Two series of copolyesters were synthesized via melt polycondensation by controlling the polymerization temperature under optimized polymerization conditions. The polymerization procedure is shown in Figure 1. The physical properties of copolyesters are strongly dependent on their chain structure and sequence, including the molecular weight and composition. The molecular weights of copolyesters were measured using GPC (Figures S1–S3). As listed in Table 1, the average molecular weight (*M*_w_) ranged from 31,000 to 45,000 g/mol and the polydispersity (PDI = *M*_w_/*M*_n_) from 1.80 to 1.94, as expected for the synthetic method used. The chemical structure of the copolyester was analyzed using the FT-IR spectrum; Figures S4 and S5 show the characteristic ester bands (1729 cm^−1^ for C=O; 1276 cm^−1^ for C-O-C) and furan heterocycles (3117 cm^−1^ for C-H, 1575 cm^−1^ for C=C stretching, and 1229 cm^−1^ for =C-O-C=) [41], indicating that the copolyester was successfully synthesized.

**Figure 2:**
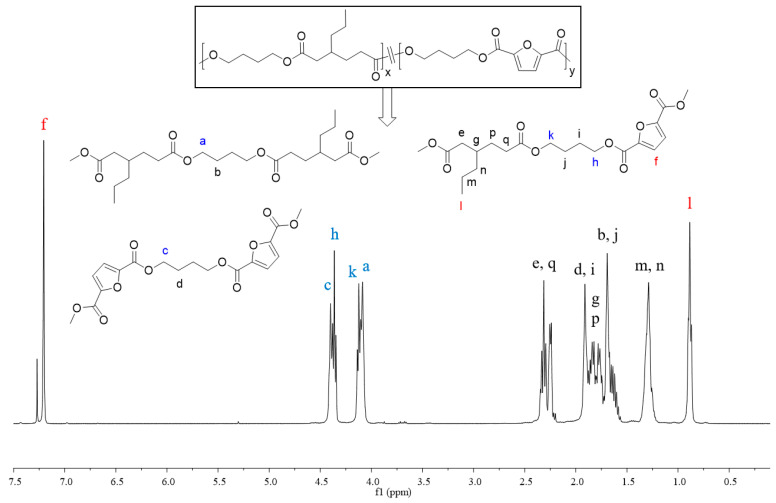
^1^H NMR spectrum and chain structures of PBA_p_F_45_, the letters a–n, p and q represent the corresponding proton and signal peak locations.

In order to better understand the sequence structure of the polymer chain, the molecular structures of copolyesters were characterized using the ^1^H NMR spectrum (Figures S6–S18). All signals correctly corresponded with the resonances of protons in different chemical environments, demonstrating the successful synthesis of the desired copolyesters. Taking the ^1^H NMR spectrum of PBA_p_F_45_ as an example, as shown in Figure 2, the two different dicarboxylic acids displayed their typical characteristic peaks; the shift appearing at 7.21 ppm (f) belonged to the furan ring of FDCA and that at 0.89 ppm (l) belonged to the CH_3_ units of 3PAA. The chemical compositions, i.e., the molar ratios of the two repeating units BDO-3PAA (BA_p_) and BDO-FDCA (BF), were calculated using the ratio of the integrals of the two resonance signals appearing at l and f. As shown in Table 1, the molar fractions of FDCA (TPA) in the products were generally close to those in the feed. The chemical shifts of -CH_2_- in BDO were close to the different ester bonds produced by the copolymerization, and different chemical shifts appeared at 4.09 ppm (a), 4.14 ppm (k), 4.36 ppm (h), and 4.40 ppm (c), respectively. According to literature reports [42], the number-average sequence length (L) of BF and BA_p_ units and the randomness (R) can be calculated using Equations (1)–(3). The integrated intensities of the partial peaks (a, k, h, and c) were calculated using the multipeaks fitting function in MestReNova software (software version number is 5.3.1-4696). Randomness is used for the description of a statistical copolymer in which the probability of finding a given monomer unit at any site in the chain is independent of the nature of the neighboring units; it should represent unity for an ideal random copolymer [43]. As shown in Table 1, the number-average sequence lengths of the copolyesters were around 1.35–4.33; therefore, they hardly formed the relatively large crystals with sharp melting peaks. The randomness of copolyesters converged to the value of 1; that is, the distribution obeyed Bernoullian statistics. If R < 1, =1, =2, and =0, it indicated that the polymers were block copolymers, random copolymers, alternating copolymers, and homopolymers, respectively [36,44]. Therefore, the synthesized copolyesters were completely random.

(1) $$L_{n,BA}=L_{n,{BA}_{p}}=1+\frac{2I_{a}}{I_{h}+I_{k}}$$

(2) $$L_{n,BF}=L_{n,BT}=1+\frac{2I_{c}}{I_{h}+I_{k}}$$

(3) $$R_{PBA_{p}F}=\frac{1}{L_{n,BF}}+\frac{1}{L_{n,BA_{p}}}$$

### 2.2. Thermal Properties

The corresponding data for the thermal stability of the copolyesters are shown in Figure S19a–c and Table 2. Typically, these materials are thermally stable up to 290 °C based on their corresponding *T*_d,5%_. The thermal properties of the copolyesters were analyzed using DSC and the corresponding curves are shown in Figure S19d–k. The glass transition temperature (*T*_g_) is the lowest temperature at which a molecular chain segment can move, and its level is directly related to the flexibility of the molecular chain. That is, the lower the *T*_g_, the greater the flexibility of the molecular chain. The higher the *T*_g_, the greater the rigidity of the molecular chain. With the addition of co-aromatic monomers, the *T*_g_ value of the copolyester steadily increased, which indicated that the flexibility of the molecular chain decreased. Different from PBA, the propyl-branched PBA_p_ was amorphous and showed that the *T*_g_ was only −58.01 °C. The introduction of a propyl group significantly reduced the crystallinity of the PBA_p_ chain, thus making flexible chains compared with rigid PBA chains. With the introduction of PBF hard segments, the *T*_g_ of the copolymer continuously increased. All copolyesters with only one *T*_g_ indicated that they were all random copolymers, which was in agreement with the results of the ^1^H NMR spectrum.

It has been reported that many papers have elucidated the origin of the double melting peak, and the accepted opinion is recrystallization [45]. The lower melting peak arises from original crystals, while the higher one arises from recrystallization during heating. The multiple melting behavior of PBA is caused by temperature-induced polymorphism, phase transformation, and melt/recrystallisation during DSC scanning [46]. The multiple melting behavior of PBT exhibits two different crystallinities (an unstrained *α*-crystal formed with a *T*_m_ of 221.63 °C and a strained *β*-crystal formed with a *T*_m_ of 215 °C). For PBAF_45_ and PBA_p_F_45_, neither melt crystallization in the cooling scan nor cold crystallization and melting in the second heating scan were observed, which indicated that they were nearly amorphous. When the content of FDCA (TPA) in the copolyester was 60–75 mol%, a greater number of BF (BT) units in the backbone chains led to the formation of larger and more regular PBF (PBT) crystalline regions; therefore, the Tm value of the copolyester increased with an increase in the FDCA (TPA) content. Melt crystallization was observed in the cooling scan curves (75.31 °C and 64.11 °C) for PBAF_75_ and PBA_p_F_75_, and cold crystallization was observed in the second heating scan (73.15 °C and 77.05 °C). These results support the interpretation of the effect of the PBA_p_ propyl group on the crystalline properties of the copolymers. With an increase in the PBA_p_ content, the copolyesters changed from semi-crystalline to amorphous and tended to be a better thermoplastic elastomer.

### 2.3. Crystal Structures

**Figure 3:**
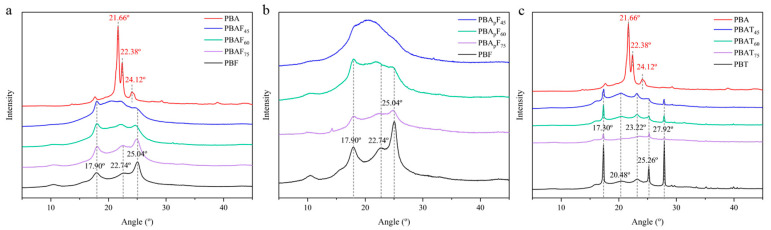
(**a**) The XRD patterns of PBA, PBF and PBAF_s_. (**b**) The XRD patterns of PBF and PBA_p_F_s_. (**c**) The XRD patterns of PBA, PBT and PBAT_s_.

The XRD patterns of PBA, PBT, PBF, PBA_p_F_s_, PBAF_s_, and PBAT_s_ are given in Figure 3. Consistent with the literature, PBA showed three main diffraction peaks at 21.66°, 22.38°, and 24.12°, which could be assigned to the (110), (020), and (021) reflection planes of an a-form crystal [43,47,48]. PBT showed five diffraction peaks at 17.30°, 20.48°, 23.22°, 25.26°, and 27.92°, which could be assigned to the (011), (010), (101), (100), and (111) reflection planes [49,50]. PBT showed multiple diffraction peaks with relatively strong intensities, indicating the regular packing of PBT chains in the crystalline phase and relatively high crystallinity. PBF showed three relatively weak diffraction peaks at 17.90°, 22.74°, and 25.04° [22,30]. Referring to relevant literature reports [42], the PBA_p_ segment with the propyl branch proved to be amorphous and showed no diffraction peaks compared with PBA.

BA, BT, and BF units are all crystallizable, and the reduced crystallinity of a copolyester is due to the randomization of the main chain structure and the incompatibility in the crystal lattices of the two components. Regarding furan-based polyesters (PBAF_s_; PBA_p_F_s_), it was found that PBAF_75_, PBAF_60_, PBA_p_F_75_, and PBA_p_F_60_ exhibited diffraction peaks that could be assigned to PBF, indicating that they had the same crystal structure as PBF. As the FDCA content decreased, PBAF_45_ and PBA_p_F_45_ both showed patterns typical of an amorphous polymer. In PBA_p_F_s_, the intensity of the diffraction peaks decreased as the BA_p_ content increased but still appeared at the same position, indicating that the branched BA_p_ segment showed no significant effect on the crystal form of the PBF segment. Influenced by the amorphous PBA_p_ unit, PBA_p_F_45_ showed no diffraction peaks in its XRD pattern (the introduction of a branched PBA_m_ chain reduced the regularity of the main chain). Regarding PBAT_s_, the three copolyesters showed the same diffraction patterns as PBT homopolymers, indicating that they had the same crystal structure as PBT. The reduction in intensities of the diffraction peaks and the broad peaks in the diffraction profiles at around 20.48° with a decrease in the BT content implied an increase in the relative proportion of the amorphous phase to the crystalline phase, which was in good agreement with previous findings [43]. In summary, the copolyesters ranged from semi-crystalline polymers to nearly amorphous polymers, which confirmed the DSC results.

### 2.4. Mechanical Properties

To better understand the composition dependence of the physical properties of the copolyesters, dumbbell-shaped samples (25 mm × 4 mm × 2 mm) were prepared by melt-casting and the tensile properties of the samples were measured by tensile testing at room temperature. Table 3 shows the tensile strength, elongation at break, and Young’s modulus of the copolyesters. When the aromatic unit content of the copolyester was 45 mol%, it had a lower tensile modulus and higher elongation. With an increase in the aromatic unit content, the tensile strength of the copolyester increased but the elongation at break decreased. This was consistent with the thermal properties and crystal structure of the copolyester observed using DSC and XRD. The tensile properties of PBAF_s_ and PBAT_s_ were consistent with those reported in the literature [40]; the low-strain property (i.e., Young’s modulus) of PBAT_s_ was higher but the high-strain property (i.e., tensile strength) was comparable for the two copolyesters. The ultimate tensile strength of PBAF_s_ was comparable with that of PBAT_s_, suggesting that the strain-induced chain orientation and the lateral alignment compensated for the secondary chain regularity of PBAF_s_. However, when comparing the moduli of PBAF_s_ and PBA_p_F_s_, we observed that the modulus of PBAF_s_ was always higher than that of PBA_p_F_s_. It is conceivable that PBA_p_F_s_ with branches had a lower packing density because branched points produce greater free volume. Considering the large elongation at break of PBA_p_F_s_, this suggests that PBA_p_F_s_ formed a structure similar to elastomers in which crystallites were well dispersed within the amorphous matrix region. In particular, the elongation at break of PBA_p_F_45_ (1865%) had a much greater value than that of PBAF_45_ (1250%). Thus, the branched polymer showed much better toughness than the linear polymers, i.e., the branching incorporated into the linear polymer increased the melt strength and conferred tension-hardening properties onto the polymer, which helped to enhance the elongation of the polymer [51].

**Figure 4:**
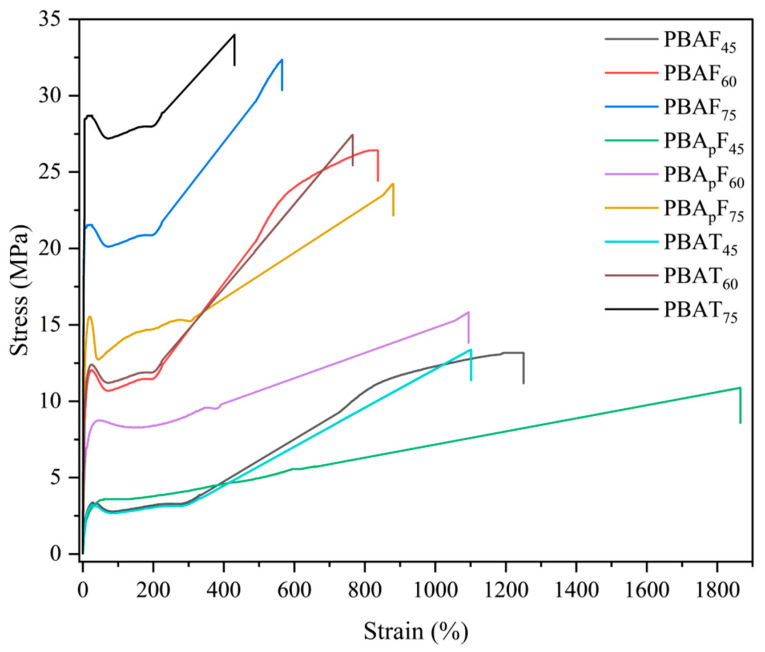
Stress–strain curves of copolyesters with different aromatic contents.

In the stress–strain curves (Figure 4), the copolyester was observed to decrease in tensile strength at the yield point and then elongate. This indicated that the synthesized copolyesters had tough ductile mechanical properties. The curves of the copolyesters with 60 mol% and 75 mol% aromatic units showed the characteristics of semi-crystalline polymers, exhibiting the following three regions: linear viscoelasticity, neck region, and strain hardening. The strain hardening was obvious, and was caused by strain-induced crystallization and chain alignment. At a 45 mol% aromatic unit content of the copolyesters, the curves showed the characteristics of amorphous polymers, which exhibit high elastic deformation. The stress–strain curve of PBA_p_F_45_ was a typical elastic curve, with a large elongation at break but without yielding behaviors, and the deformation was reversible to some extent.

**Figure 5:**
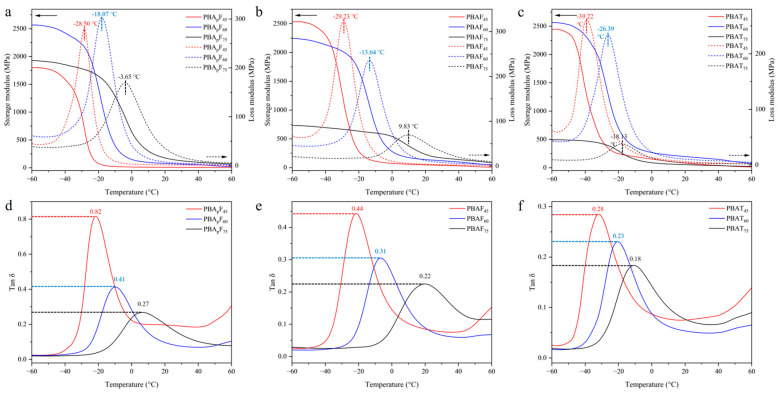
Dynamic mechanical properties of copolyesters. (**a**–**c**) the changes in the storage modulus and loss modulus of copolyesters at different temperatures. (**d**–**f**) the changes in the tan *δ* of copolyesters at different temperatures.

The dynamic mechanical properties of the copolyesters were studied using DMA. The test samples were rectangular film samples (35 mm × 5 mm × 2 mm) prepared by melt-casting. Figure 5 shows the changes in the storage modulus, loss modulus, and tan *δ* of the copolyesters at different temperatures, where tan *δ* is the ratio of the loss modulus to the storage modulus. According to literature reports [52], a DMA curve can provide three values for the glass transition temperature, which can be analyzed as the peak maximum of the loss modulus (*E″*), the peak maximum of the loss factor (tan *δ*), or the onset temperature of a decrease in the storage modulus (*E′*) (Table S1). Each of these temperatures has its physical merit and interpretation [53]. In this study, the *T*_g_ was defined as the temperature corresponding with the peak maximum of the loss modulus (*E″*). In random copolyesters, the *T*_g_ tends to increase with an increase in the aromatic unit content, which was consistent in this study (Figure 5a–c), further proving that the copolyesters were random copolyesters. An increase in the temperature from −60 °C to the *T*_g_ resulted in a large decrease in the storage modulus, which was due to the polymer segment undergoing micro-Brownian motion in the amorphous regions when the temperature increased to the *T*_g_.

### 2.5. Rheological Properties

**Figure 6:**
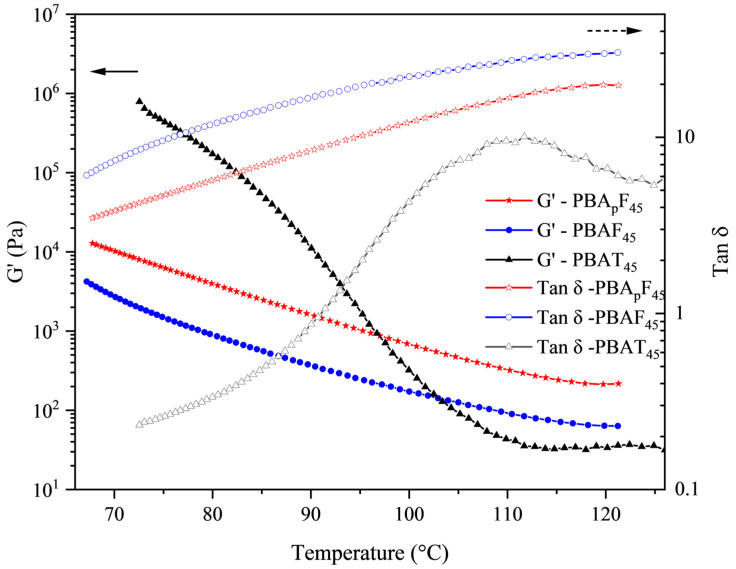
Rheological measurements in a dynamic mode; *G′* and tan *δ* as a function of temperature.

Figure 6 shows the rheological properties of PBA_p_F_45_, PBAF_45_, and PBAT_45_ as a function of temperature (the rheological property curves of the other polymers are shown in Figures S20 and S21). The storage modulus (*G′*) of the three samples rapidly decreased with an increase in temperature. It should be noted that PBA_p_F_45_ had a higher storage modulus (*G′*) compared with PBAF_45_ over the entire temperature range observed. The rheological properties were mainly affected by the following three factors [40]: the chemical structure of the chain (chain rigidity), the size of the chain (molecular weight), and the degree of entanglement (physical network). The GPC test results showed that the molecular weight difference between PBAF_45_ and PBA_p_F_45_ was negligible (Table 1) and the rheological parameters were generally proportional to the chain rigidity. However, we observed the opposite trend, which suggests that PBA_p_F_45_ had a stronger physical network. Typically, physical networks are created by either a heterostructure or a spatial entanglement of the chains [54]. The literature reports that heterostructures can be determined by evaluating the yield stress, which represents the minimum energy required for the collapse of the heterostructure [55]. It was clear from the tensile test results that the yield stress values for both polymers were close to zero (Figure 4), implying only a small amount of inhomogeneity in the molten state. Therefore, the higher *G′* of PBA_p_F_45_ compared with PBAF_45_ could fully be attributed to higher number of chain entanglements. It is conceivable that PBA_p_F_s_ with branches had a lower packing density because the branch points generated greater free volume, which could interdiffuse and entangle with the aromatic segments more efficiently.

### 2.6. Enzymatic Degradation

**Figure 7:**
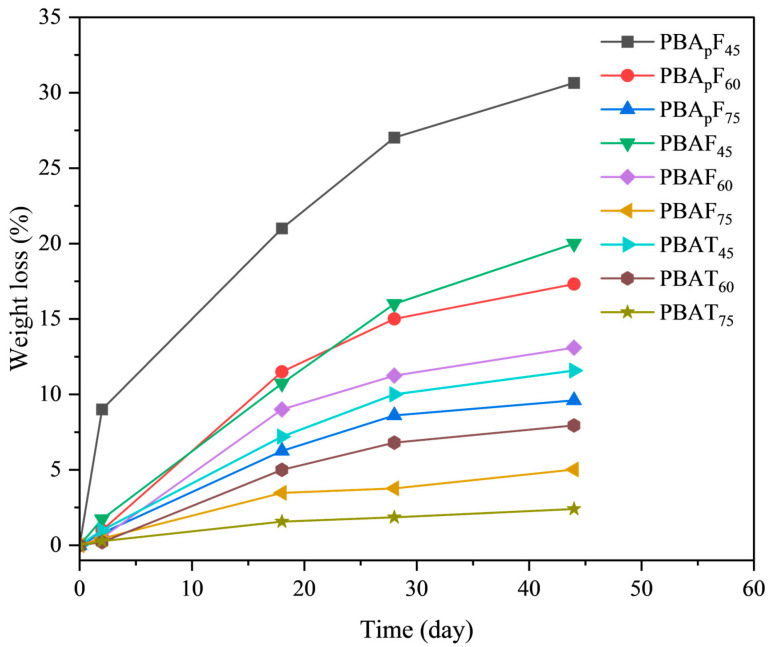
Weight loss during enzymatic degradation of copolyesterase.

It is necessary to study the enzymatic degradation of copolyesters when developing novel bio-based biodegradable materials. In this paper, the enzymatic degradation of the copolyesters using porcine pancreatic lipase was detected in a phosphate buffer solution at 37 °C. As shown in Figure 7, the degradation speed strongly depended on the copolymer composition; the greater the aromatic units, the slower the degradation. For copolyesters PBA_p_F_45_, PBA_p_F_60_, PBAF_45_, PBAF_60_, and PBAT_45_, the weight loss linearly increased with time within 45 days of enzymatic degradation, and the maximum weight losses were 30.7, 17.3, 19.8, 12.7, and 11.6%, respectively. For PBAF_75_ and PBAT_75_, the maximum weight loss was less than 5.0%, which indicated that they could not be degraded by porcine pancreatic lipase, which was consistent with the data reported in the literature under the same enzymatic hydrolysis conditions [39].

The faster the degradation of PBAF_s_ and PBA_p_F_s_ than PBAT_s_, which was due to the inherent structural irregularity of FDCA, the more hydrophilic and oxygen-containing the furan ring, enhancing the chemical affinity between the polar active site of the enzymes and the copolyester chains. A planar TPA with a benzene ring exhibits tight stacking of polymer chains, which seriously hinders degradation [40]. Compared with PBAF_s_, the relatively high biodegradation rate of PBA_p_F_s_ could be explained by the introduction of branched 3PAA, which reduces the crystalline state and melting point of copolyesters. A lower crystallinity is more conducive to the adhesion and erosion of enzymes, so enzyme degradation is more likely to happen. With an increase in 3PAA, the degradation rate of PBA_p_F_s_ gradually accelerated. The movement and relative conformational change in the polymer chain segment could be explained using DMA (Figure 5), and the peak position of the loss tangent (tan *δ*) indicated the glass transition temperature (*T*_g_) of the polymer. The peak intensity of the loss tangent of PBA_p_F_45_ (0.82) was almost twice that of PBAF_45_ (0.44); that is, the mobility of the chain segment was enhanced, which provided better coordination with the enzyme and improved the enzymatic selectivity.

## 3. Experimental Procedure

### 3.1. Materials

Adipic acid (AA, 99%), 1,4-dicarboxybenzene (TPA, 99%), and tetrabutyl titanate (TBT, 97%) were purchased from the Aladdin Reagent Company (Shanghai, China). Lipase from porcine pancreas (30–90 units/mg) was purchased from the Sigma-Aldrich Company (St. Louis, MO, USA). Polystyrene standards were purchased from Waters Asia Limited (Shanghai, China). 3-Propyladipic acid (3PAA, 99%) is a self-made reagent derived from lignin. 1,4-Butanediol (BDO, 99%) and 2,5-furandicarboxylic acid (FDCA, 99%) are derived from cellulose. The other reagents (methanol, dichloromethane, etc.) were of ACS reagent grade and used without further purification unless otherwise noted.

### 3.2. Synthesis of Copolyesters

A series of copolyesters were synthesized using petrochemical and bio-based monomers via a two-step melt polymerization method (esterification and polycondensation) in a high-pressure autoclave. The synthesized copolyesters included poly(butylene adipate-co-terephthalate) (PBAT), poly(butylene adipate-co-furanoate) (PBAF), and poly(butylene 3-propyladipate-co-furanoate) (PBA_p_F).

Typically, according to the molar percentage of FDCA in the feed, PBA_p_F was labeled as PBA_p_F_45_, PBA_p_F_60_, and PBA_p_F_75_. For the synthesis of PBA_p_F_45–75_, FDCA, 3PAA, BDO (diol/diacid molar ratio of 2:1), and TBT (0.6 wt%, relative to the total of diacids) were added into a high-pressure autoclave equipped with mechanical stirring. The reactor was evacuated several times and nitrogen was injected to remove oxygen. In the first step, the reaction was conducted at 180 °C under a nitrogen flow and stirred at 400 rpm for 12 h. In the second step, a vacuum was slowly applied to 20 Pa to avoid excessive foaming and to minimize oligomer sublimation, which is a potential problem during melt polycondensation [56]. The temperature was slowly increased to 200 °C while the stirring speed was also increased to 600 rpm. The reaction was conducted at 200 °C for 3 h under a reduced pressure of 20 Pa. Other copolyesters were synthesized using the same procedure.

### 3.3. Characterization

Gel permeation chromatography (GPC): The molecular weight was determined using GPC. The instrument was equipped with a Waters 2414 refractive index detector (Milford, MA, USA) and an Agilent PLgel 5 μm MIXED-C (made in GB) column (Santa Clara, CA, USA). Chloroform was used as an eluent at a flow rate of 1 mL/min, the column temperature was fixed at 35 °C, and polystyrene standards were used for calibration.

Fourier transform infrared spectroscopy (FT-IR): FT-IR (Thermo Scientific IS-5, Waltham, MA, USA) was used with a universal attenuated total reflection (ATR) accessory. The measurements were made at a resolution of 6 cm^−1^ from 600 cm^−1^ to 4000 cm^−1^ and with 32 scans.

Nuclear magnetic resonance (NMR): ^1^H NMR spectra were recorded using a Bruker Ascend 400 MHz NMR spectrometer (Faellanden, Switzerland) at 400 MHz. Chloroform-d (CDCl_3_) and tetramethylsilane (TMS) were used as the solvent and internal standard, respectively. Due to the poor solubility of PBAT_75_ and PBF in CDCl_3_, trifluoroacetic acid-d (CF_3_COOD) was used as the solvent.

X-ray diffraction (XRD): The crystal morphology of the copolyesters was analyzed using XRD analyses with a Bruker D8 focus, using Cu-Ka radiation in the scan ranging from 5° to 60° in 2θ with steps of 0.02° at a fixed counting time of 4 s.

Thermogravimetric analysis (TGA): TGA was conducted using a TGA55 (TA Instruments, Shanghai, China). Samples were heated from 35 °C to 600 °C at a heating rate of 10 °C/min under a N_2_ atmosphere (flow rate: 20 mL/min).

Differential scanning calorimetry (DSC): DSC was conducted using a DSC25 (TA Instruments). The sample was placed in an Al_2_O_3_ crucible and a heat/cool/heat procedure was used to avoid the influences of thermal histories. We used a heating/cooling rate of 10 °C/min between −80 °C and 300 °C under a N_2_ atmosphere (flow rate: 35 mL/min).

Rheological testing: The rheological properties of the copolyesters were analyzed using a rotational rheometer and a Haake Mars 40 rheometer (Thermo Fisher Scientific) with a plate geometry of 20 mm. Rheological measurements were obtained in dynamic mode, with *G’* and tan *δ* as a function of temperature (the test temperature was based on the melting point of the sample as a reference). The details of the test are as follows: measuring geometry, P20/Ti-02190837; damping, 30.00; gap, 1.500 mm; γ_0_, 0.5000%; f, 1.000 Hz; t, 2040.00 s; T, 122.00 °C–20.00 °C.

Tensile testing: The mechanical properties of the copolyesters were measured using a universal testing machine (UTM 8104, Jinan Wenteng Testing Instrument Co., Ltd., Jinan, China). Samples of 25 mm × 4 mm × 2 mm were prepared by melt-casting using a dumbbell-shaped mold. Then, the samples were stretched at 25 °C at a 50 mm/min stretching rate. Each sample was tested at least 5 times and the average values were reported.

Dynamic mechanical analysis (DMA): The dynamic mechanical properties of the samples were analyzed using a PerkinElmer DMA 8000 (Waltham, MA, USA) at the temperature range of −60 to 100 °C and a heating rate of 3 °C/min. All the measurements were performed in tension mode at fixed frequencies of 1 Hz and at 0.1% strain. The specimens were prepared in the shape of a rectangular bar with dimensions of 35 mm × 5 mm × 2 mm by melt-casting using a square mold.

### 3.4. Biodegradation Analysis

The copolyester was biodegraded by lipase from porcine pancreas. The specimens were prepared in the shape of rectangular films with dimensions of 20 mm × 20 mm × 0.3 mm by melt-casting using a square mold. The films were placed in a phosphate buffer solution (pH = 7.2) with 0.3 mg/mL lipase at 37 °C. We regularly removed the film and weighted it until a constant weight was reached. The degree of biodegradation was estimated according to the weight loss [39].

## 4. Conclusions

In conclusion, two series of copolyesters were synthesized by melt polycondensation by controlling the polymerization temperature under optimized polymerization conditions. The physical properties of copolyesters are strongly dependent on their chain structure and sequence, including their molecular weight and composition. The molecular structures of the copolyesters were characterized using the ^1^H NMR spectrum. The number-average sequence lengths of the copolyesters were around 1.35–4.33 and they hardly formed relatively large crystals with sharp melting peaks. The randomness of the copolyesters converged to the value of 1; that is, the distribution obeyed Bernoullian statistics. Therefore, the synthesized copolyesters were completely random.

Considering the large elongation at break of PBA_p_F_s_, this suggested that PBA_p_F_s_ formed a structure similar to elastomers in which crystallites were well dispersed within the amorphous matrix region. In particular, the elongation at break of PBA_p_F_45_ (1865%) had a much greater value than that of PBAF_45_ (1250%). Thus, the branched polymer showed much better toughness than linear polymers, i.e., the branching incorporated into the linear polymer increased the melt strength and conferred tension-hardening properties onto the polymer, which helped to enhance the elongation of the polymer. The stress–strain curve of PBA_p_F_45_ was a typical elastic curve, with a large elongation at break but without yielding behaviors, and the deformation was reversible to some extent. The dynamic mechanical properties of the copolyesters were studied using DMA. An increase in the temperature from −60 °C to the *T*_g_ resulted in a large decrease in the storage modulus, which was due to the polymer segment undergoing micro-Brownian motion in the amorphous regions when the temperature was increased to the *T*_g_. The relatively high biodegradation rate of PBA_p_F_s_ could be explained by the introduction of branched 3PAA, which reduced the crystalline and melting point of the copolyesters. A lower crystallinity is conducive to the adhesion and erosion of enzymes, so enzyme degradation was more likely to happen. In all, a novel lignocellulose-based PBA_p_F could find end applications as a biodegradable elastomer or impact modifier for other polymers, which not only have the potential to partially replace petroleum polymers but also have unique advantages and superior performance.

## Figures and Tables

**Table 1:** Molecular weights, chemical composition, sequence lengths (L), and degree of randomness (R) of copolyesters.

	GPC			^1^H NMR				
Sample	*M*_n_	*M*_w_	PDI	FDCA or TPA mol% in Copolyesters		Sequence Length ^b^		R ^c^
				Feed	Calculated ^a^	L_n,BAp_ (L_n,BA_)	L_n,BF_ (L_n,BT_)	
PBA_P_F_45_	22,986	41,419	1.80	45	43.01	1.84	1.61	1.16
PBA_P_F_60_	17,021	31,567	1.85	60	58.14	1.45	2.28	1.13
PBA_P_F_75_	18,709	35,114	1.88	75	70.42	1.35	4.33	0.97
PBAF_45_	24,616	44,902	1.82	45	39.92	1.79	1.64	1.17
PBAF_60_	19,834	38,003	1.92	60	54.20	1.49	2.29	1.11
PBAF_75_	16,167	37,504	1.94	75	68.49	1.27	3.72	1.06
PBAT_45_	-	-	-	45	42.96	2.51	1.60	1.02
PBAT_60_	-	-	-	60	57.72	1.93	1.92	1.04
PBAT_75_	-	-	-	75	69.08	1.65	2.35	1.03

^a^ The molar ratio of copolyesters was determined by the integration of ^1^H NMR spectra. ^b^ Calculated according to Equations (1) and (2). ^c^ Calculated according to Equation (3).

**Table 2:** Thermal properties of polyesters.

Sample	TGA			DSC			
	*T*_d,5%_ ^a^ (°C)	*T*_d,max_ ^b^ (°C)	R_600_ ^c^ (%)	Second Heating			Cooling
				*T*_m_ (°C)	*T*_cc_ ^d^ (°C)	*T*_g_ (°C)	*T*_c_ ^e^ (°C)
PBA_p_	305	371	0.995	-	-	−58.01	-
PBA	332	403	0.966	56.24	-	-	32.42
PBF	336	366	6.6	165.77	-	31.22	120.70
PBT	289	392	5.384	221.63	-	46.01	197.87
PBA_p_F_45_	333	386	3.765	-	-	−30.32	-
PBA_p_F_60_	331	377	5.902	113.02	-	−17.74	-
PBA_p_F_75_	335	377	8.136	134.99	77.05	−2.84	64.11
PBAF_45_	344	385	5.593	-	-	−28.69	-
PBAF_60_	342	380	8.280	115.22	78.50	−12.06	-
PBAF_75_	340	377	8.529	139.33	73.15	8.41	75.31
PBAT_45_	353	397	3.982	103.85	64.51	−40.30	67.80
PBAT_60_	315	394	5.024	135.45	-	−27.09	108.36
PBAT_75_	290	393	4.398	151.68	-	−19.08	124.29

^a^ Temperature at which 5% weight loss was determined in the TGA. ^b^ Temperature of maximum degradation rate. ^c^ Remaining weight at 600 °C. ^d^ Cold crystallization temperature. ^e^ Melt crystallization temperature measured by DSC.

**Table 3:** Tensile strength (*σ*), elongation at break (*ε*), and Young’s modulus (*E*) of copolyesters.

Sample	*σ*_max_ (MPa)	*ε*_max_ (%)	*E* (MPa)
PBAF_45_	13.17 ± 0.15	1250 ± 6	31.45 ± 0.26
PBAF_60_	26.42 ± 0.19	837 ± 5	190.26 ± 2.24
PBAF_75_	32.35 ± 0.13	565 ± 2	404.69 ± 5.68
PBA_p_F_45_	10.88 ± 0.17	1865 ± 11	22.64 ± 0.33
PBA_p_F_60_	15.83 ± 0.23	1095 ± 5	55.34 ± 2.57
PBA_p_F_75_	24.22 ± 0.14	881 ± 3	309.08 ± 3.10
PBAT_45_	13.37 ± 0.08	1101 ± 5	55.17 ± 2.43
PBAT_60_	27.44 ± 0.15	765 ± 3	228.76 ± 3.58
PBAT_75_	34.00 ± 0.12	430 ± 3	412.01 ± 6.85

## Data Availability

Available data are presented in the manuscript.

## Supplementary Materials

The following supporting information can be downloaded at: https://www.mdpi.com/article/10.3390/molecules30040878/s1, Figure S1: The molecular weights of copolyesters PBA_p_F. Figure S2: The molecular weights of copolyesters PBAF. Figure S3: The molecular weights of copolyesters PBAT. Figure S4: FT-IR spectrum of copolyesters. Figure S5: FT-IR spectrum of copolyesters. Figure S6: ^1^H NMR spectrum and chain structures of PBA_p_. Figure S7: ^1^H NMR spectrum and chain structures of PBA. Figure S8: ^1^H NMR spectrum and chain structures of PBF. Figure S9: ^1^H NMR spectrum and chain structures of PBT. Figure S10: Structure and characteristic peaks of copolyesters. Figure S11: ^1^H NMR spectrum of PBA_p_F_60_. Figure S12: ^1^H NMR spectrum of PBA_p_F_75_. Figure S13: ^1^H NMR spectrum of PBAF_45_. Figure S14: ^1^H NMR spectrum of PBAF_60_. Figure S15: ^1^H NMR spectrum of PBAF_75_. Figure S16: ^1^H NMR spectrum of PBAT_45_. Figure S17: ^1^H NMR spectrum of PBAT_60_. Figure S18: ^1^H NMR spectrum of PBAT_75_. Figure S19: a–c TGA curves of copolyesters recorded from 30 to 600 °C at 10 °C/min under N_2_ atmosphere, and DSC curves of the first cooling d–f and the second heating g–k scans at the corresponding heating/cooling rates of 10 °C/min under a N_2_ flow of 20 mL/min. Table S1: Glass transition temperature provided by DMA analysis. Figure S20: Rheological measurements in a dynamic mode, *G′* and tan *δ* as a function of temperature. Figure S21: Rheological measurements in a dynamic mode, *G′* and tan *δ* as a function of temperature.
