# Supplementary material for: Synthesis of Lignocellulose-Based Poly(Butylene 3-Propyladipate-Co-Furanoate): Replacing Adipate
Source: Molecules. 2025 Feb 14;30(4):878. doi: 10.3390/molecules30040878 (PMC11857895; doi:10.3390/molecules30040878)
Supplement: Supplementary file 1 [file molecules-30-00878-s001.zip › molecules-3392158-supplementary.pdf]

Supplementary information

Synthesis of lignocellulose-based poly(butylene 3-propyladipate-co-furanoate): replacing adipate

Ruijia Hu [1,*], Weihao Li [1], Xi Zhou [1], Shunmin Yi [2], Xingfu Zheng [1], Qiufeng Mo [1] and Wanyu Liu [1,*]

[1] Guangxi Key Laboratory of Advanced Microwave Manufacturing Technology, Guangxi Academy of Sciences, Nanning 530007, China; ruijiahu@gxas.cn (H.R); lwh17877259373@163.com (L.H); zx_gxkx@163.com (Z.X); zhengxingfu@gxas.cn (Z.X.); 18078131362@163.com (M.Q); liuwanyu11@gxas.cn (L.W)

[2] Guangxi Key Laboratory of Advanced Structural Materials and Carbon Neutralization, Guangxi Colleges and Universities Key Laboratory of Environmental-Friendly Materials and Ecological Restoration, School of Materials and Enviroment, Guangxi Minzu University, Nanning 530105, China; 20200040@gxmzu.edu.cn (S.Y.)

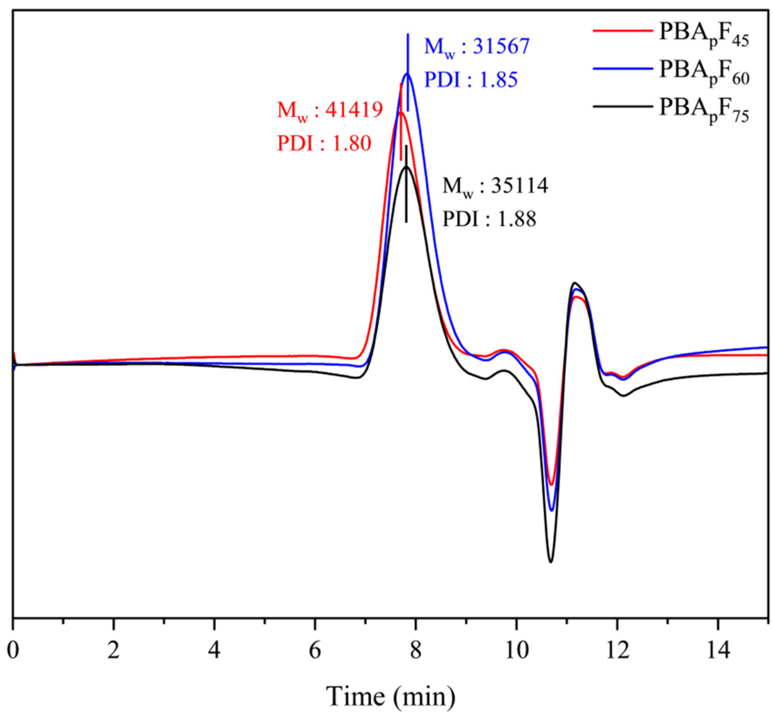

Fig. S1 The molecular weights of copolyesters PBA$_P$F.

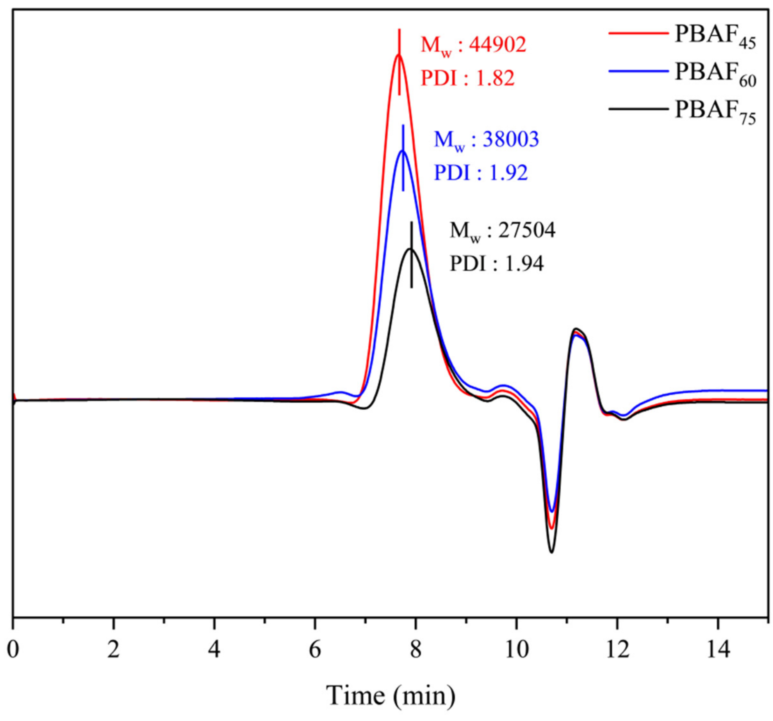

Fig. S2 The molecular weights of copolyesters PBAF.

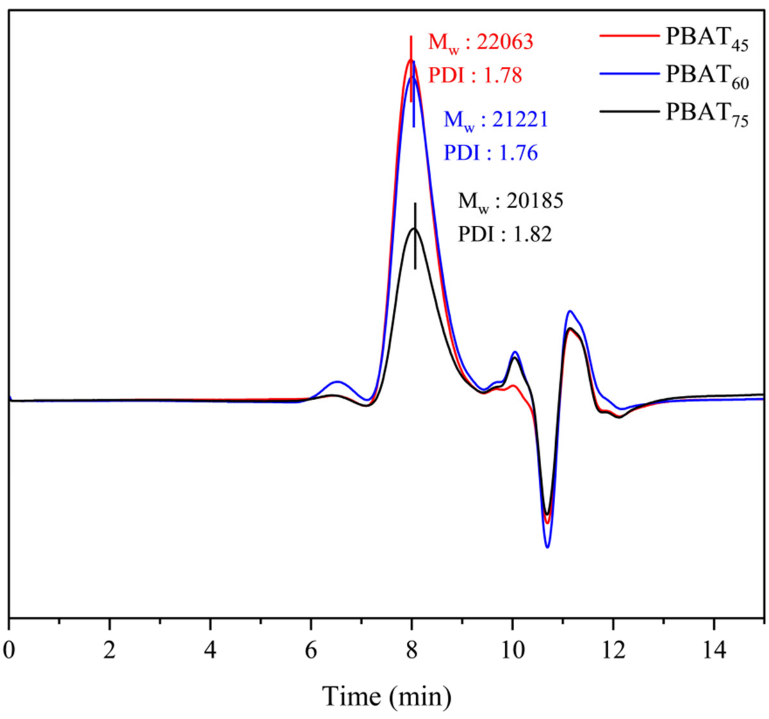

Fig. S3 The molecular weights of copolyesters PBAT.

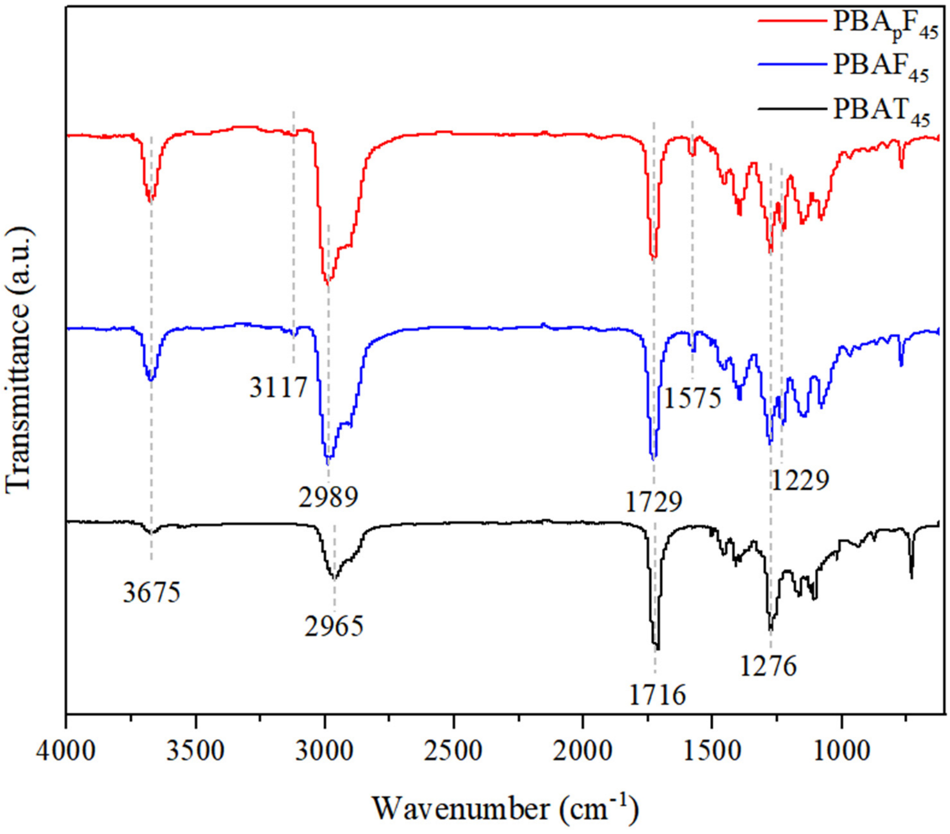

Fig. S4 FT-IR spectrum of copolyesters.

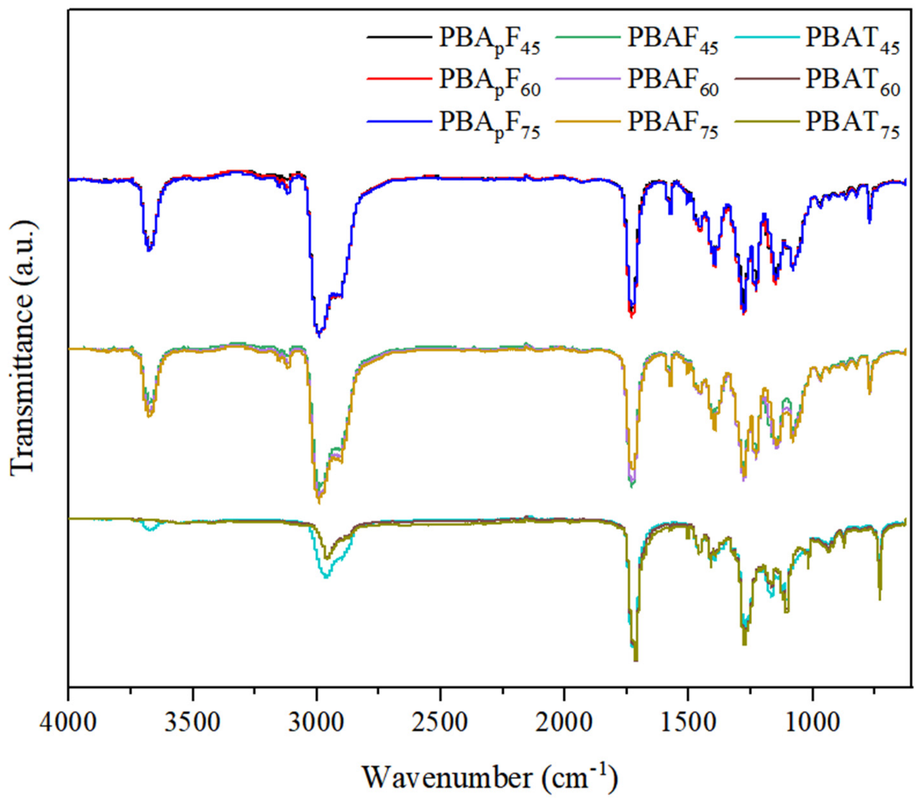

Fig. S5 FT-IR spectrum of copolyesters.

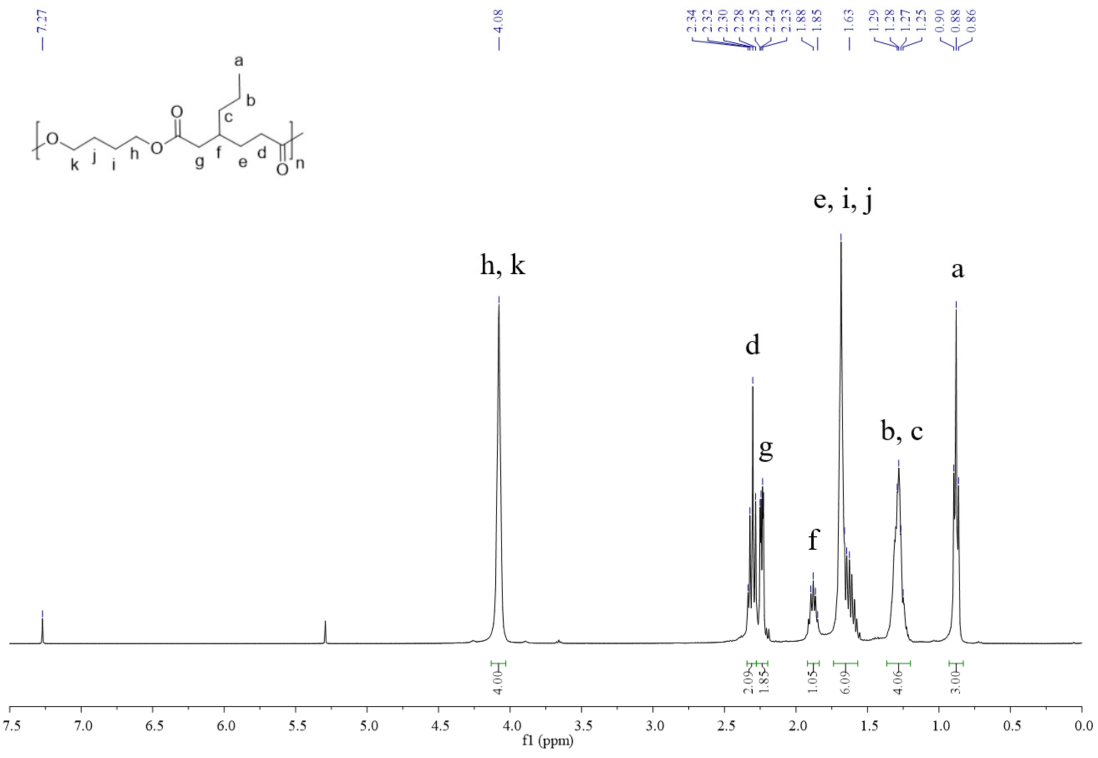

Fig. S6 ^{1}H NMR spectrum and chain structures of PBA_p.

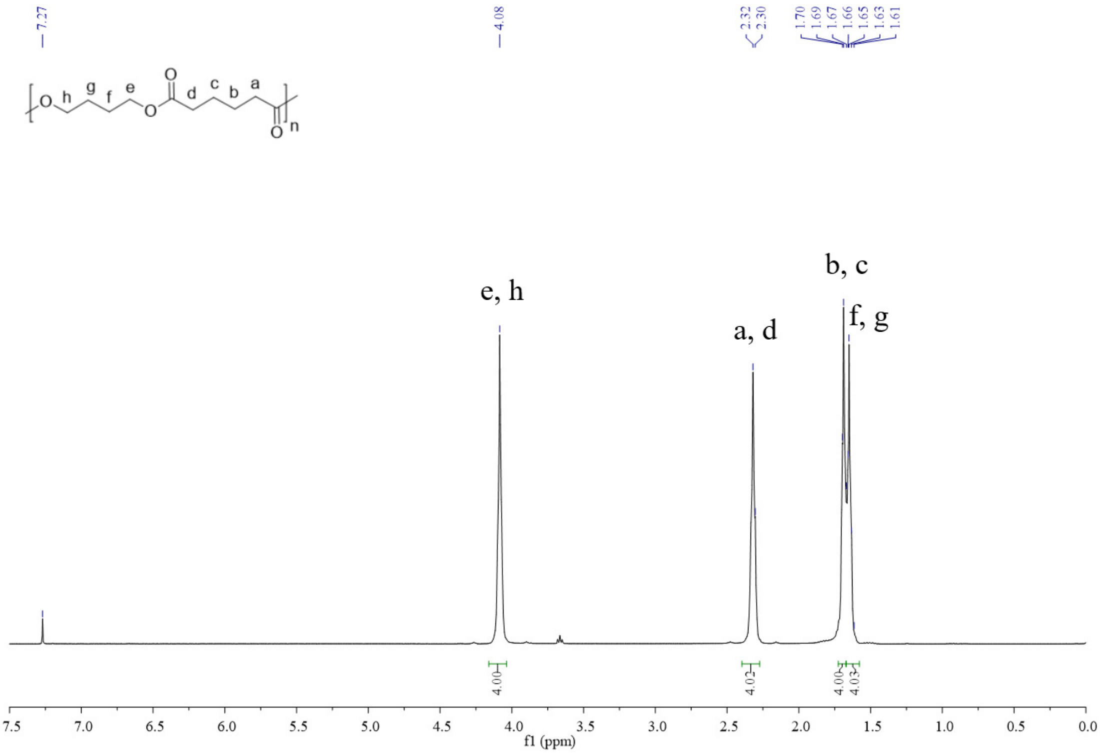

Fig. S7 ^{1}H NMR spectrum and chain structures of PBA.

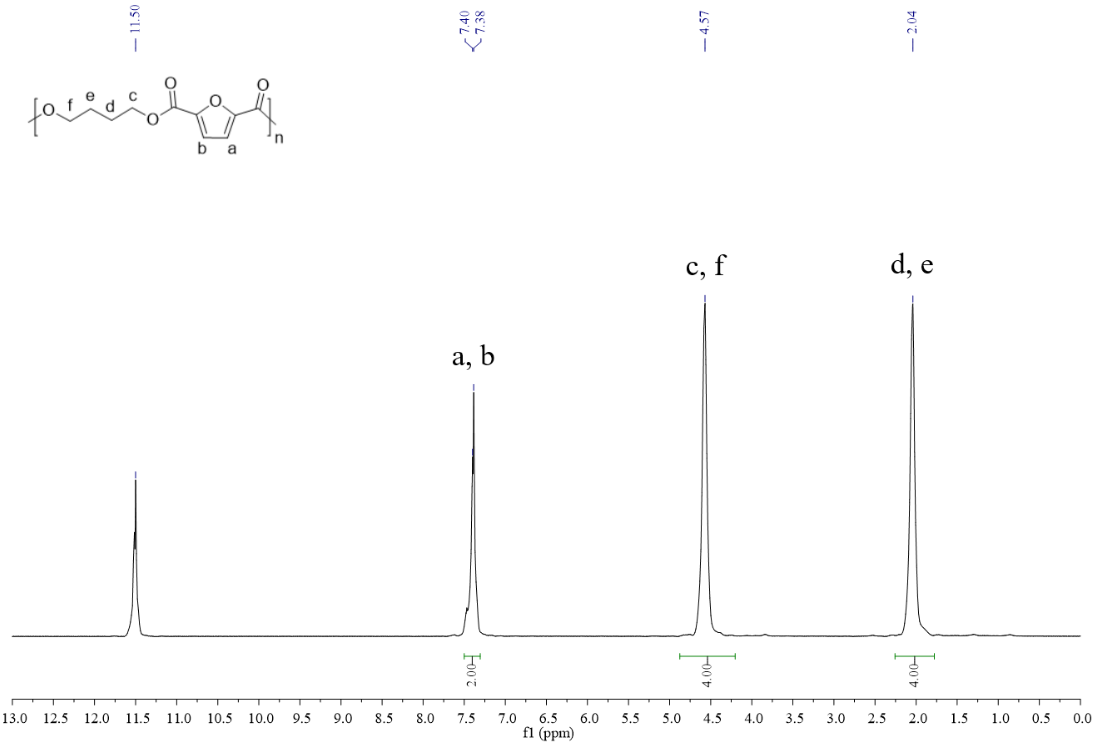

Fig. S8 ^{1}H NMR spectrum and chain structures of PBF.

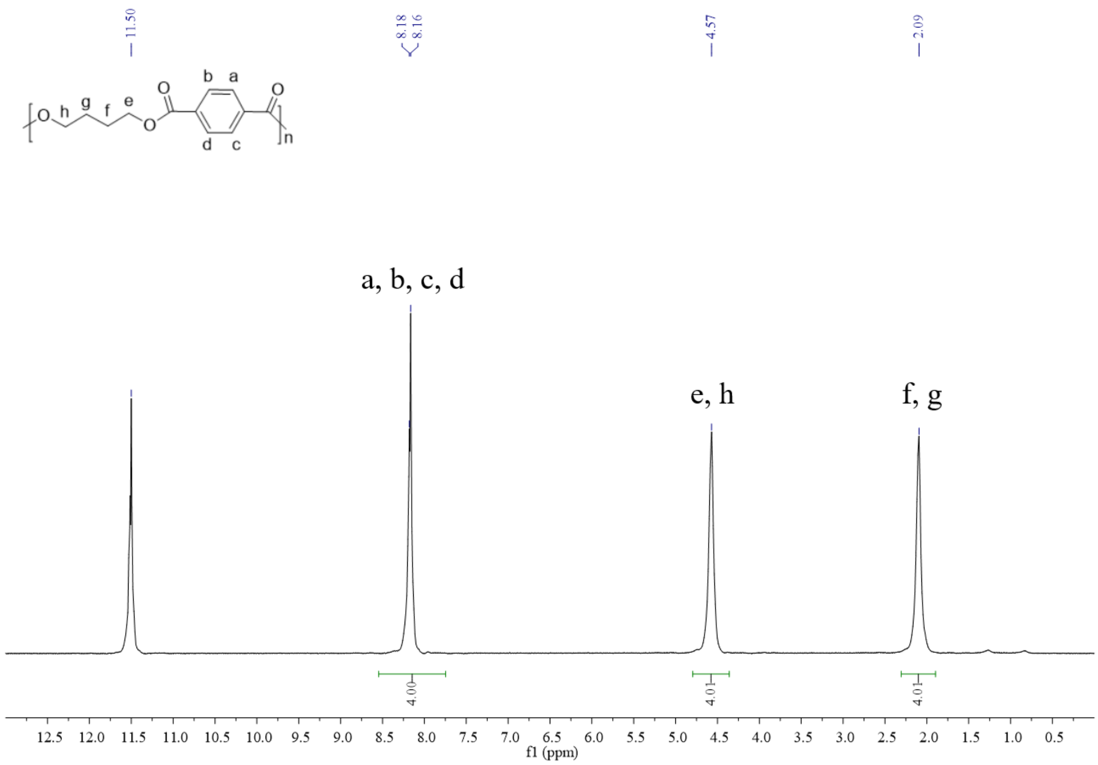

Fig. S9 ^{1}H NMR spectrum and chain structures of PBT.

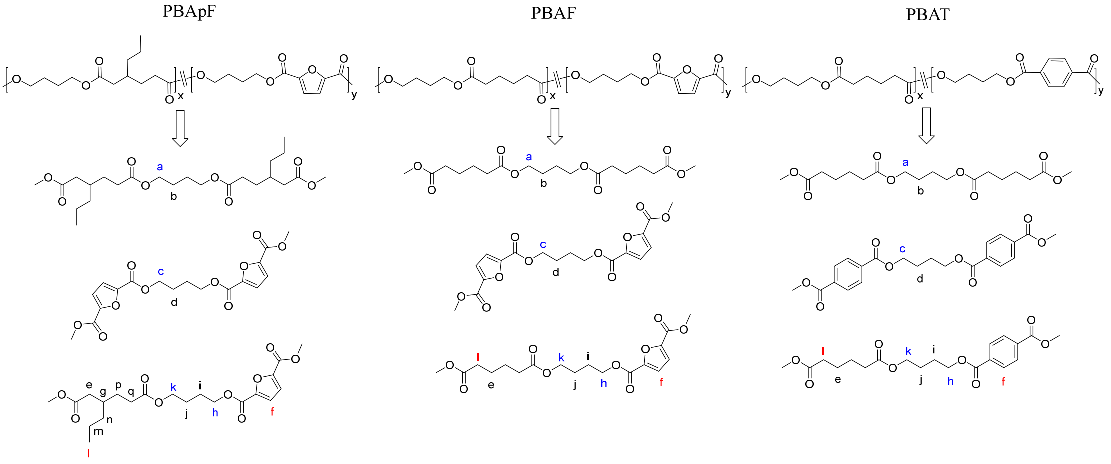

Fig. S10 Structure and characteristic peaks of copolyesters.

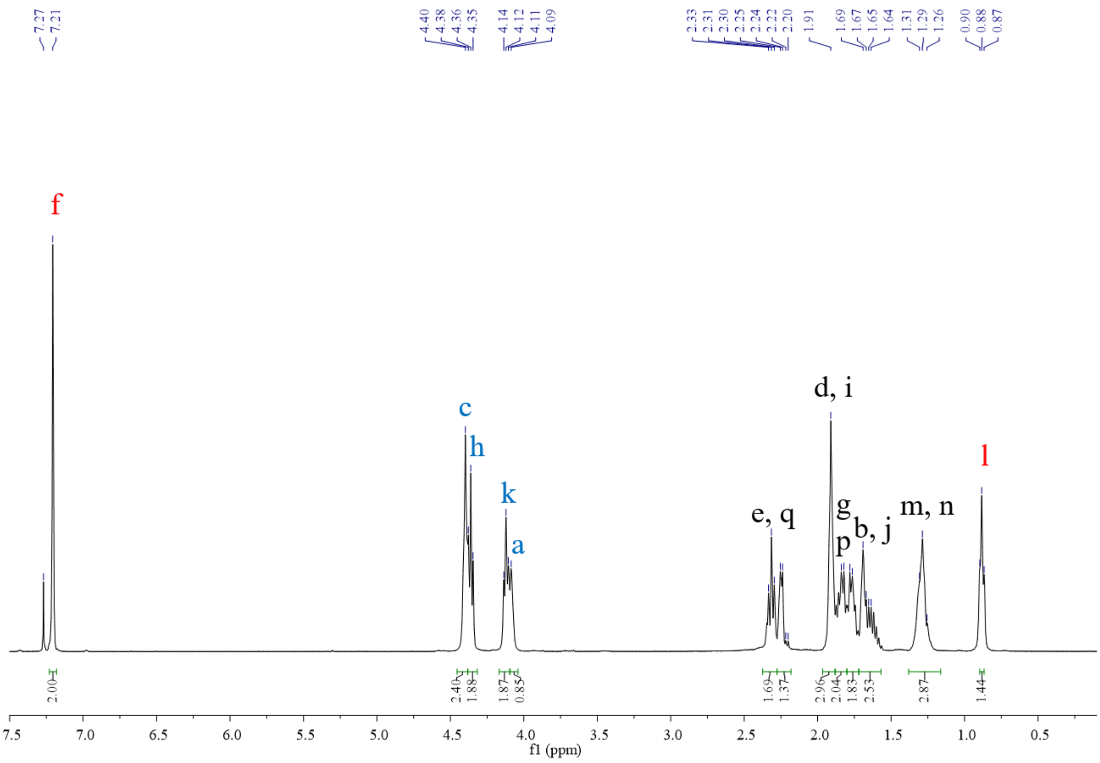

Fig. S11 ^{1}H NMR spectrum of $PBA_{P}F_{60}$.

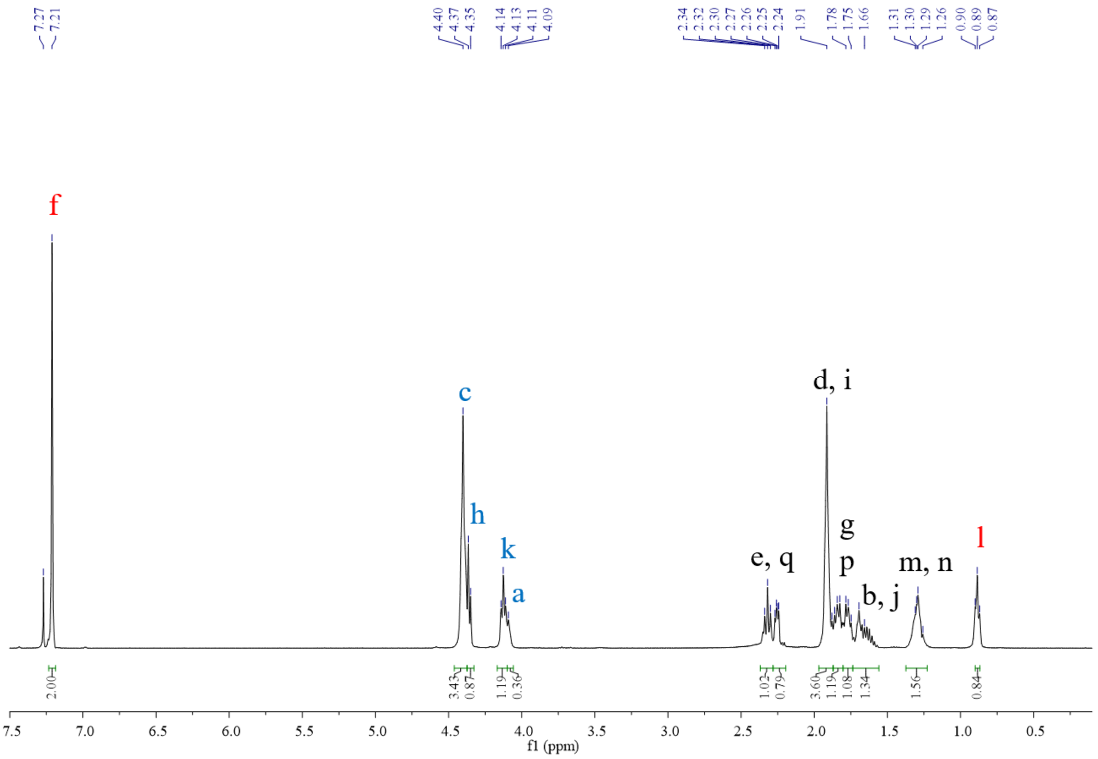

Fig. S12 ^{1}H NMR spectrum of $PBA_{P}F_{75}$.

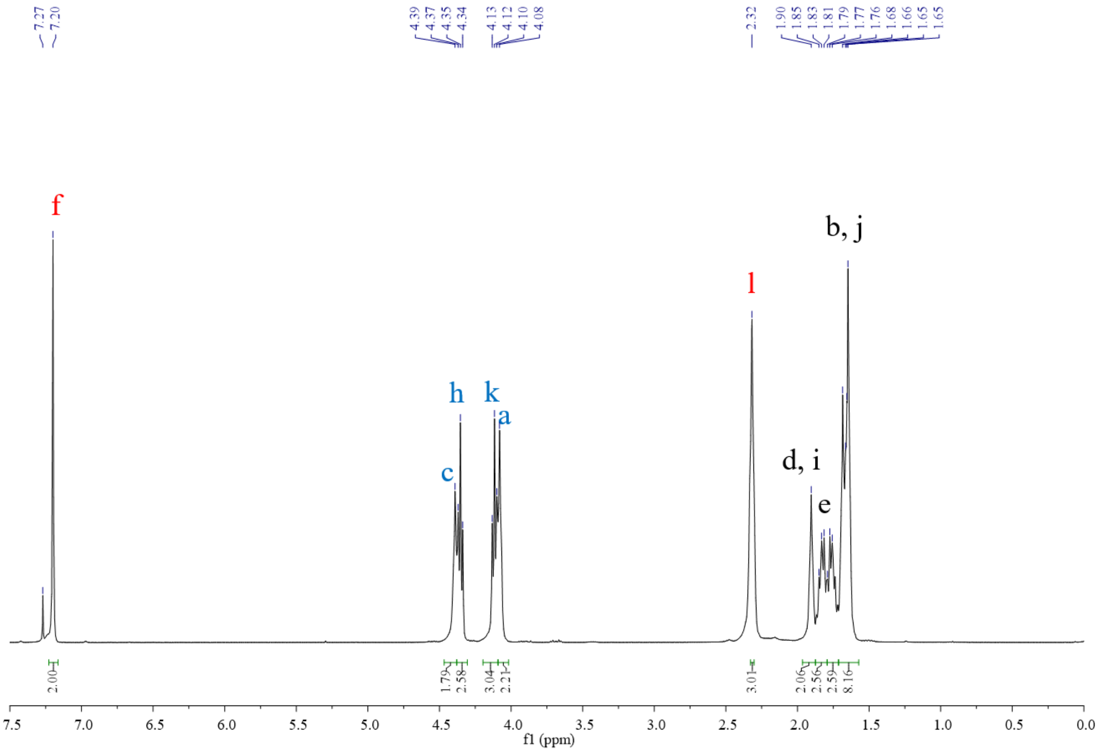

Fig. S13 ^{1}H NMR spectrum of $PBAF_{45}$.

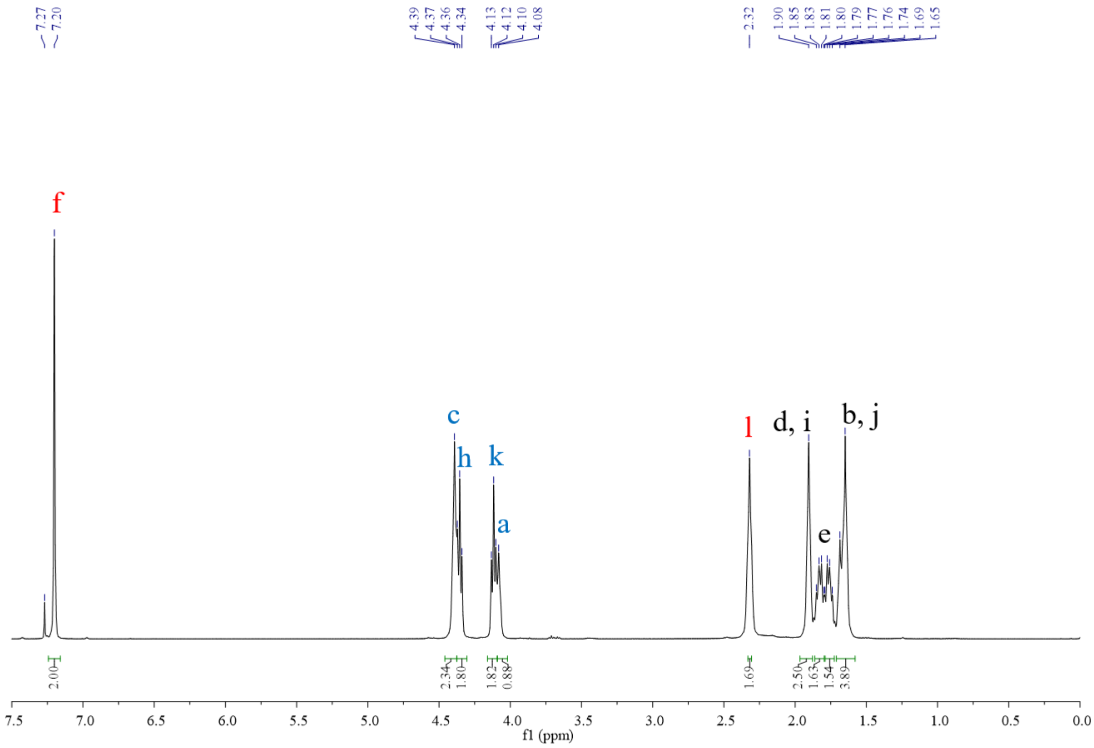

Fig. S14 ^{1}H NMR spectrum of $PBAF_{60}$.

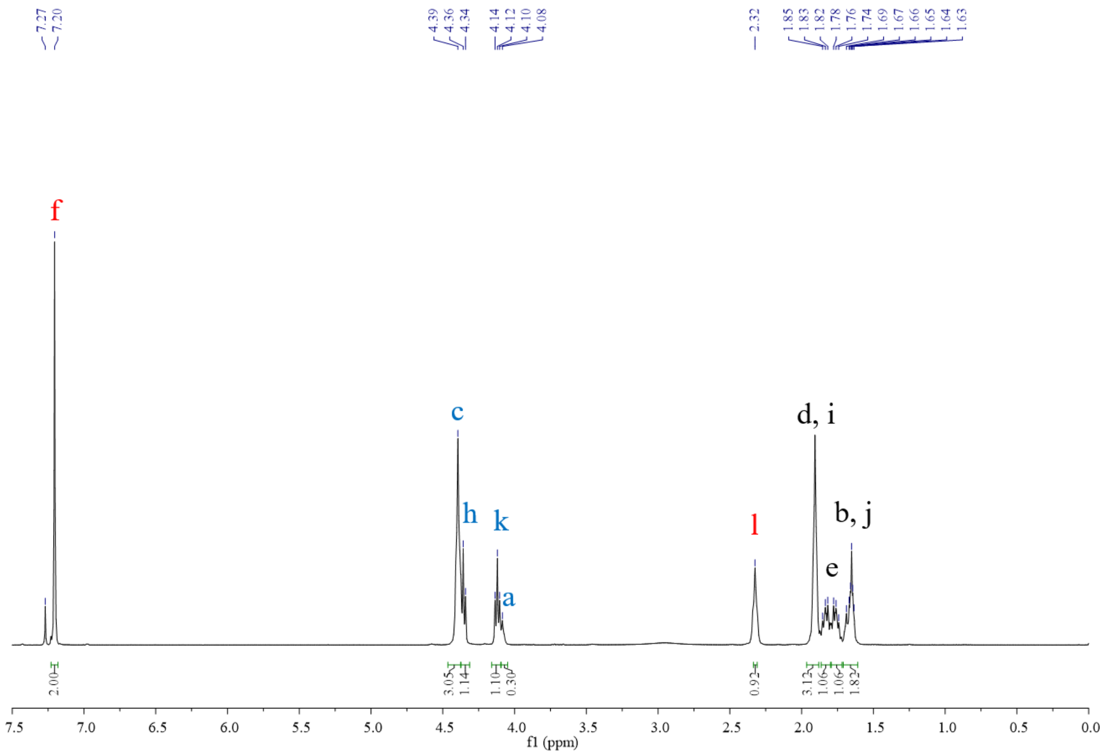

Fig. S15 ^{1}H NMR spectrum of $PBAF_{75}$.

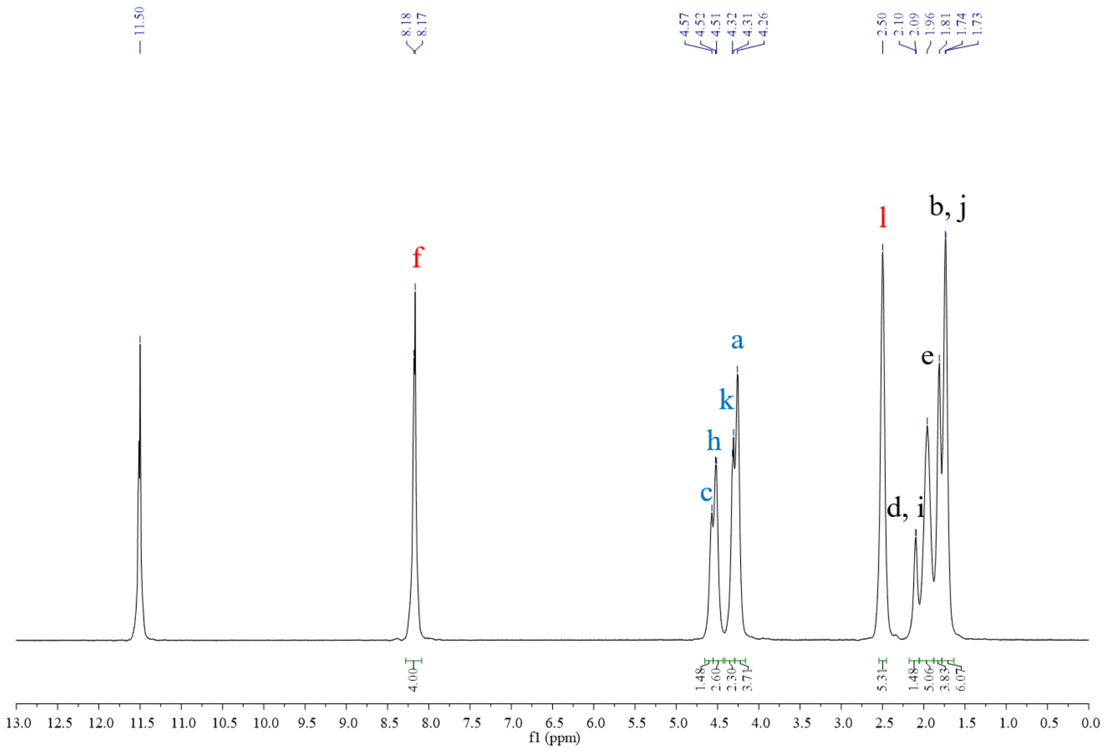

Fig. S16 ^{1}H NMR spectrum of $PBAT_{45}$.

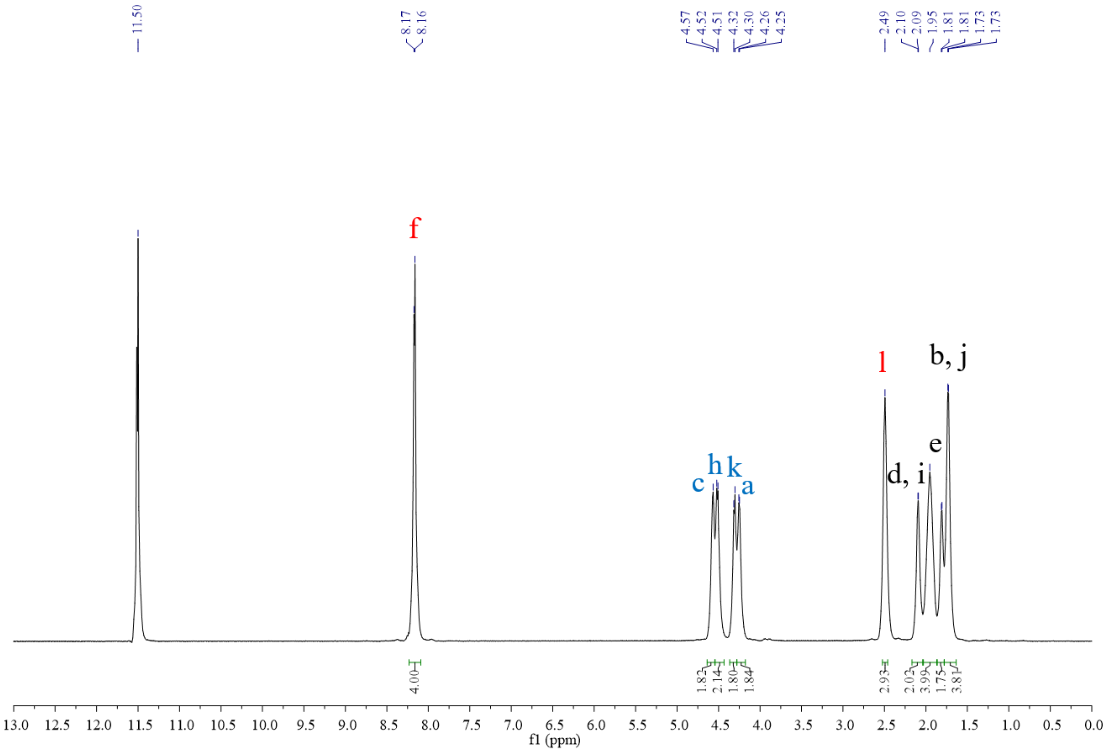

Fig. S17 ^{1}H NMR spectrum of $PBAT_{60}$.

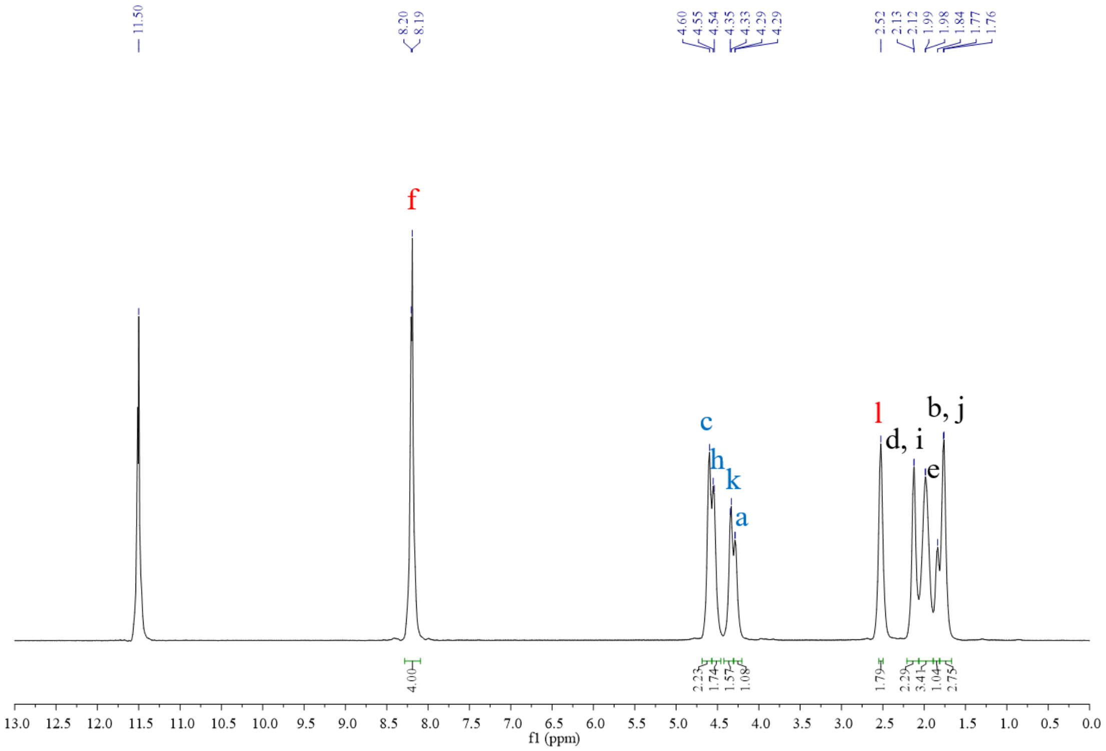

Fig. S18 ^{1}H NMR spectrum of $PBAT_{75}$.

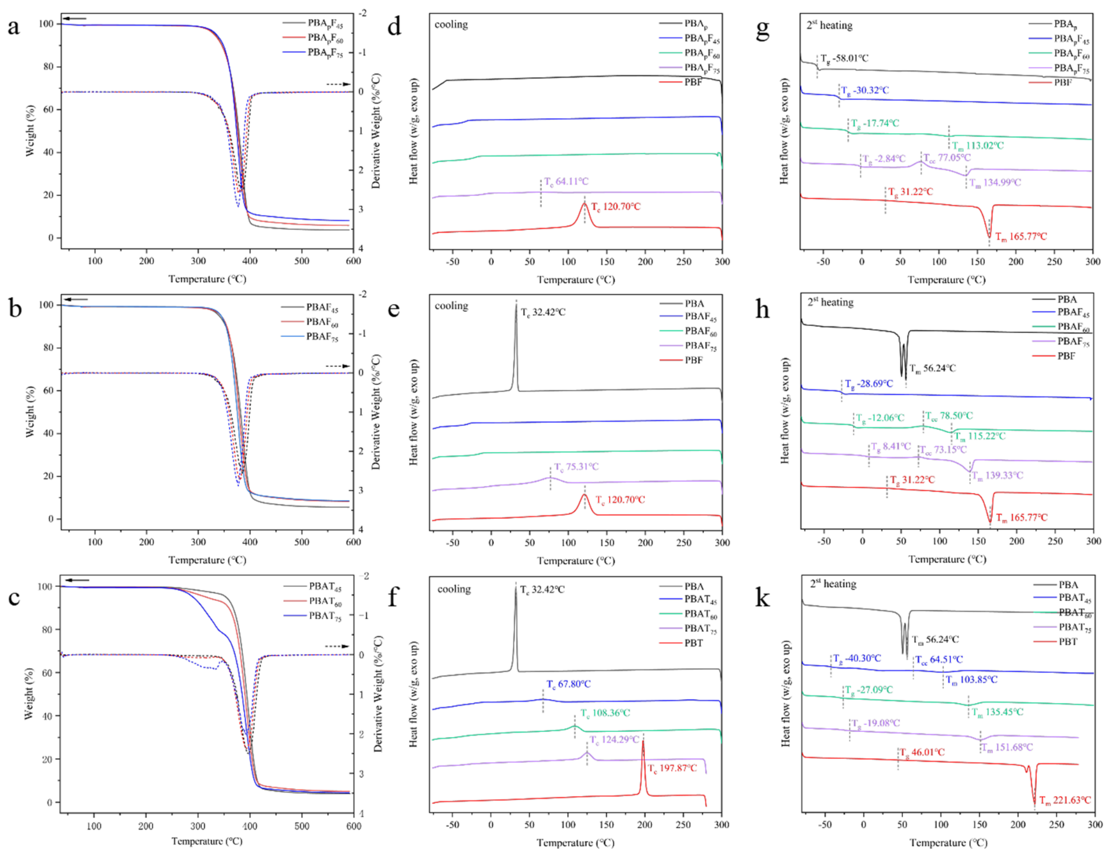

Fig. S19 a-c TGA curves of copolyesters recorded from 30 to 600 °C at 10 °C/min under N_2 atmosphere, and DSC curves of the first cooling d-f and the second heating g-k scans at the corresponding heating/cooling rates of 10 °C/min under a N_2 flow of 20 mL/min.

Table S1 Glass transition temperature provided by DMA analysis.

Sample	$T_{\tan\delta}$ ^{a}(℃)	$T_{E''}$ ^{b}(℃)	$T_{E'}$ ^{c}(℃)
PBAF$_{45}$	-21.68	-29.23	-38.11
PBAF$_{60}$	-6.70	-13.64	-22.79
PBAF$_{75}$	18.84	9.83	-0.90
PBA$_p$F$_{45}$	-21.68	-28.50	-36.72
PBA$_p$F$_{60}$	-9.72	-18.07	-27.15
PBA$_p$F$_{75}$	6.77	-3.65	-16.15
PBAT$_{45}$	-31.76	-39.22	-47.86
PBAT$_{60}$	-20.80	-26.39	-33.80
PBAT$_{75}$	-11.01	-18.13	-34.08

^{a} Temperature at peak maximum of loss modulus (E''); ^{b} Temperature at peak maximum of loss factor (tan δ); ^{c} Onset temperature of decrease of storage modulus (E').

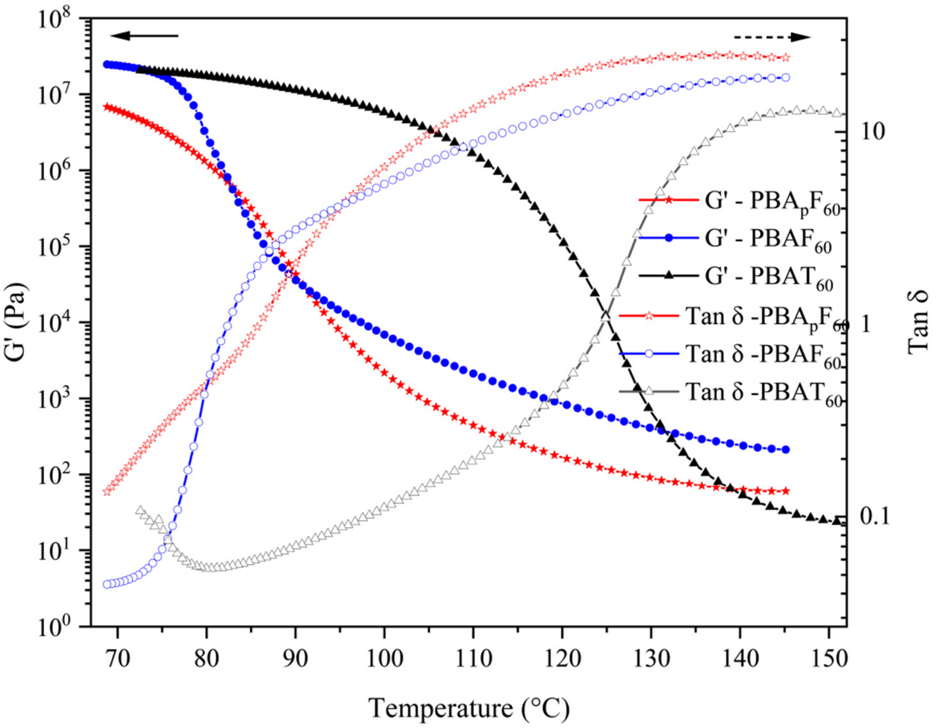

Fig. S20 Rheological measurements in a dynamic mode, *G′* and tan *δ* as a function of temperature.

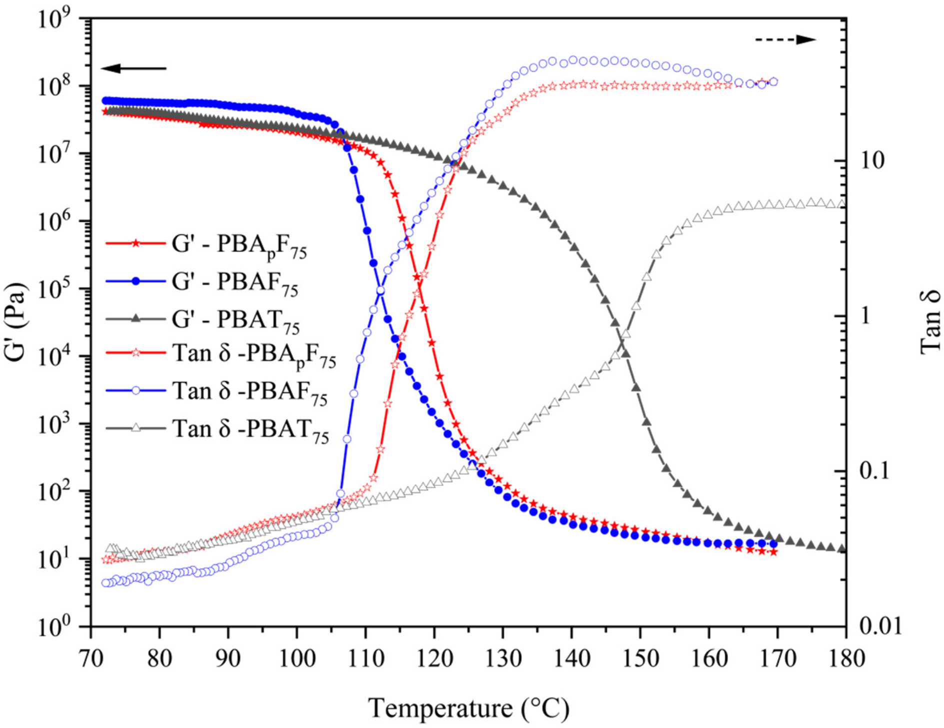

Fig. S21 Rheological measurements in a dynamic mode, *G′* and tan *δ* as a function of temperature.
